# Evaluation of a new community-based curriculum in disaster medicine for undergraduates

**DOI:** 10.1186/s12909-016-0746-6

**Published:** 2016-08-26

**Authors:** Nidaa Bajow, Ahmadreza Djalali, Pier Luigi Ingrassia, Luca Ragazzoni, Hussein Ageely, Ibrahim Bani, Francesco Della Corte

**Affiliations:** 1 CRIMEDIM - Research Centre in Emergency and Disaster Medicine and Computer Science Applied to Medical Practice, University of Eastern Piedmont, Novara, Italy; 2 Disaster Medicine Unit, Mohammad Bin Naif Medical Center, King Fahd Security College, P O Box 89489, Riyadh, 11682 Saudi Arabia; 3 Medical School of Jazan University, Jazan, Saudi Arabia

**Keywords:** Curriculum, Disaster medicine, Saudi Arabia

## Abstract

**Background:**

Nowadays, many medical schools include training in disaster medicine in undergraduate studies. This study evaluated the efficacy of a disaster medicine curriculum recently designed for Saudi Arabian medical students.

**Methods:**

Participants were 15 male and 14 female students in their fourth, fifth or sixth year at Jazan University Medical School, Saudi Arabia. The course was held at the Research Center in Emergency and Disaster Medicine and Computer Sciences Applied to the Medical Practice in Novara, Italy.

**Results:**

The overall mean score on a test given before the course was 41.0 % and it increased to 67.7 % on the post-test (Wilcoxon test for paired samples: z = 4.71, *p* < 0.0001). There were no significant differences between the mean scores of males and females, or between students in their fourth, fifth or sixth year of medical school.

**Conclusions:**

These results show that this curriculum is effective for teaching disaster medicine to undergraduate medical students. Adoption of this course would help to increase the human resources available for dealing with disaster situations.

## Background

The last two decades have seen a growing interest in disaster medicine [1–5]. This is understandable given the number and magnitude of natural disasters (such as the 2004 Indian Ocean earthquake and consequent tsunami, and hurricane Katrina) as well as man-made and technological disasters (such as the 9/11 bombing, the Fukushima Daiichi nuclear disaster and the ongoing series of worldwide terrorist attacks).

Disaster medicine, which is pivotal in such circumstances, has been traditionally a postgraduate form of training. However, in the face of large-scale disasters, the personnel trained in disaster medicine can be overwhelmed by the demands of the disaster and other medical personnel have to pitch in. In view of the gaps in undergraduate and postgraduate disaster medicine education, [6–10] some educational authorities have called for improved disaster medicine education [11, 12] and several medical schools and professional organizations have developed curricula in disaster medicine for education of all physicians [1–4, 13–17].

A survey of all Saudi Arabian medical schools [18] showed that teaching of disaster medicine was scarce, but there was willingness to institute such training at the undergraduate level, with a preference for both didactic and interactive learning activities coupled with a combination of on-site education and distance e-learning. Consequently, a training curriculum contextualized to the local environmental and socioeconomic milieu was developed [19]. At the same time, because crises can span geographic and political regions, the program is also consistent with international educational and disaster medicine standards [20, 21]. The aim of the current study was to evaluate the efficacy of this curriculum in improving the knowledge of Saudi Arabian medical students.

## Methods

### Setting and participants

Saudi medical students are routinely sent abroad during the summer for further education or training, and the community-based disaster medicine course was piloted at the Research Center in Emergency and Disaster Medicine and Computer Sciences Applied to the Medical Practice (CRIMEDIM) in Novara, Italy. The 2-week course started on 16 June 2014.

The participants were 15 male and 14 female students in their fourth, fifth or sixth year at Jazan University Medical School, Saudi Arabia, which agreed to participate in this study. The students were selected on the basis of having a minimum overall grade of 3.5/5.0 and possession of good knowledge of English.

The course was held at the Research Center in Emergency and Disaster Medicine (CRIMEDIM), which was established in 2007 as part of the University of Eastern Piedmont, Italy. The course was delivered by 19 instructors, of whom 12 were with University of Eastern Piedmont, Italy, specialized in anesthesia and disaster medicine (*n* = 7) or in disaster medicine (*n* = 3), anesthesia and humanitarian medicine (*n* = 1) or prehospital management (*n* = 1). The others were from Como Hospital, Italy, Venice University, Italy, Bunbury and Busselton Hospitals, West Australia, and Jazan University, Saudi Arabia, and specialized in disaster medicine, prehospital management, emergency medicine, and emergency and critical care.

All the students consented to full participation before the program and had not received any instruction in disaster medicine. The study was approved by the Ethics Committee of Jazan Medical School in Saudi Arabia.

### Curriculum

The development and constitution of the curriculum have been described [19]. The curriculum was developed through a five-stage approach by five international experts in collaboration with stakeholders from the Jazan area, and focus was placed on interactive, student-centered content. The curriculum introduces core principles in emergency medicine, public health, and disaster management using several approaches (lectures, workshops, simulations, group discussions, case studies, and role-playing) to promote higher cognitive engagement (Table 1). Five major domains were presented in this curriculum: (1) general concepts of disaster medicine; (2) disaster risk reduction; (3) disaster & mass casualty incident management; (4) principles of community awareness; (5) training sessions for community education.

**Table 1:** The contents and schedule of the disaster medicine course

Time	Day 1	Day 2	Day 3	Day 4	Day 5
09:00—10.30	- Course presentation - Student and faculty presentation - Pre-test	Lecture Hazard identification and risk assessment	Case study Discussion of Jeddah flood 2009	Lecture Medical aspects of different disasters Lecture & GD Prehospital disaster management	ISEE Familiarization & hospital disaster preparation
11:00—12:30	Lecture Introduction to disaster management	Workshop Using available tools to assess risks to Jazan	Lecture & GD International humanitarian law Ethics in disasters	XVR Mass casualty triage	ISEE Prehospital and hospital disaster response
13:30—15:00	Lecture Differences between emergency and disaster medicine, and humanitarian health	Lecture, EL, GD Strategies for preventing and mitigating risks to self and to others	VISIT Novara University Hospital	Lecture & GD Command and control and the incident command system	ISEE -After action review -Debriefing of the first week
15:30—17:00	Video lecture The emergency medical systems used in Jazan	Lecture, SDE, GD General concepts of adult teaching	Visit Novara EMS dispatch center	XVR Disaster scene management	
	Day 6	Day 7	Day 8	Day 9	Day 10
09:00—10.30	Video lectures - Impact & principle of community participation - Tools for community awareness & public education	Lectures & GD Top ten priorities & public health assessment during humanitarian emergencies	Lecture & RP Techniques to handle psychological reactions caused by disasters	Lecture Infectious diseases in emergencies	Lecture International coordination for disaster response
11:00—12:30	Lecture & 3 mock exercises Community education for environmental mitigation measures	Lectures & CS -Humanitarian standards in context: The Sphere Project -Community education for public health surveillance	Brainstorm Preparation of the pilot session for community education	Material preparation Group 1 simulation of a pilot session for community education	Presentation & feedback Discussion of the group pilot session
13:30—15:00	Lecture Community awareness: Role of volunteers Brainstorm Preparation of the pilot session for community education	Video lecture The role of Ministry of Health & Red Crescent during population displacement in Jazan	Trip Lake Lago Maggiore	Material preparation Group 2 simulation of a pilot session for community education	-Post-test -Debriefing & farewell
15:30—17:00	Visit Advanced medical post			Material preparation Group 3 as above	

*L* lecture, *VL* video lecture, *GD* group discussion, *WS* workshop, *RP* role-play, *ME mock EL* experiential learning, *SDE* self-diagnostic exercise, *ISEE* Interactive Simulation for Emergencies (www.inovaria.com), *EMS* Emergency Medical Service, *XVR* virtual reality training software for education, training and assessment of incident response safety professionals (www.xvrsim.com), *MOH* Ministry of Health, *Sphere Project* http://www.sphereproject.org

General concepts of disaster medicine included 13 lectures. In the introduction to disaster medicine lectures, the students received an introductory lecture on disaster medicine, including different key terms and classification and identification of the causes of disasters (man-made and natural) by two approaches: trigger events and speed onset events. In trigger events, the disaster cause can be natural or anthropic. The natural cause can be primary such as in earthquakes or secondary as in floods. Anthropic causes are divided into three subcategories: technical (as in industrial incidents and building collapses), social (as in mass gatherings) and war (conventional, or chemical, nuclear or biological bombing). The second approach is based on the speed of onset of the disaster (slow or progressive) both of them expose the students to both types of disaster (man made and natural) in the lectures of medical aspect of disaster the students focused more about direct and indirect impact of natural disasters such as floods and earthquakes. They applied the principles of disaster management cycle to the Jeddah floods. They were also introduced to the definition and level of complex humanitarian emergencies resulting from violent conflict and compared their effect with the effects of different types of natural disasters such as earthquake, floods and high winds.

In disaster risk assessment, the students identified the common hazards in their area, such as floods and earthquakes, and population displacements caused by the violent conflict in Yemen, and mass casualties from road traffic accidents. They identified and applied the safety strategies that should be followed in both conventional and nonconventional (chemical hazards) mass casualties.

In mass casualties incident management, the students gained an understanding of the different approaches to managing mass casualties, the concept and functions of an advance command post/team (ACP), and the concept of hospital disaster preparedness. They also demonstrated the common types of mass casualty triage. Virtual reality simulation was used to introduce the students to manmade disaster scenarios, such as building collapse and fire on boats at a seaport. In these simulations, they were asked to handle elements of the disaster response, including standard operation procedures for notification and confirmation of mass casualties, incident command system, triage, treatment, evacuation of victims to proper facilities, and activation of the hospital disaster response plan, as well as stabilization of the victims.

In principles of community awareness, the students performed an exercise on public health education according to the World Health Organization strategies [22–24] and three mock community education sessions to demonstrate the principles of community awareness. These included health promotion for educators in primary schools in areas affected by earthquakes, preparation of oral rehydration solution for mothers with young children in pediatric clinics in camps, and corpse disposal during a cholera outbreak.

In the last domain, training for community education, the students were oriented to the principles of adult learning to enable them to design sessions for educating communities in Saudi Arabia in disaster preparedness. After that, they were divided into groups of 3 − 5 students and asked to prepare community training activities. There were six sessions. In the first two sessions, the students chose the topics on which to prepare workshops for community education and brainstormed with instructors to define their aims, objectives, target audiences, and educational strategy. One topic they agreed on was a workshop for primary school educators about fire disasters, including the medical impact of the fire and the treatment of first and second-degree burns and suffocation. The second was on health promotion and standard precautions in primary schools during a pandemic infection, for example by coronavirus or H1N1. During the next three sessions, each group of students prepared one of the following topics: types of burns and injuries resulting from a fire disaster, the minimum first aid required to save the victims, and video lectures on activation of an evacuation plan and standard precaution procedures during a pandemic influenza. In the last session, on the last day of the program, each group of students presented their community education session and received feedback from the instructors. The post-test was administered after this session. Passing the course required a grade of ≥ 60 % in the post-test and participation in the presentation of the community training session.

### Evaluation of the efficacy of the course

A pre-test was conducted on the first day of the course and a post-test on the last day. The questions were obtained mainly from the question test banks of CRIMEDIM. The instructors were asked to prepare questions relevant to their subjects if they could not find appropriate questions in the CRIMEDIM database. Each 30-min test consisted of 25 multiple choice questions with only one correct answer. One point was given for a correct answer and zero for a wrong answer.

The questions in the two tests were different but both sets covered the four major course domains: (1) general concepts of disaster medicine, (2) disaster risk reduction, (3) mass casualty incident management, and (4) community disaster awareness. The number of questions for each domain paralleled the relative weight of the domain in the curriculum. Four experts from CRIMEDIM reviewed the pre-test and post-test.

To obtain feedback from the students about the course, they were asked to fill an evaluation form at the end of the course. In addition to the nine questions (mostly Likert scale), they were asked for suggestions on how the course might be improved.

We also sought to measure the third level of Kirkpatrick’s evaluation, [25] which specifies for behavior changes after training and education, by contacting the students by email after one and half years. Those who did not respond to the email were contacted by phone whenever possible.

### Statistical analysis

Statistical analysis was done using SPSS, version 11.0 (Statistical Package for Social Science (SPSS, 22®, IBM, NY, USA). The reliability of test items was tested by calculating Cronbach’s alpha coefficients for the knowledge and practice items.

The data are presented as mean percent score and standard deviation. The non-parametric Mann-Whitney test was used to compare the means of two groups, and the Kruskal Wallis test was used to compare the means for three groups. Wilcoxon Signed Rank test was used to compare pre-test with post-test mean scores. Level of significance was set at *p*-value ≤ 0.05.

## Results

Cronbach’s alpha coefficients for the knowledge and practice items, calculated on the pretest, were 0.723 and 0.897, respectively, demonstrating the reliability of the test items.

**Fig. 1:**
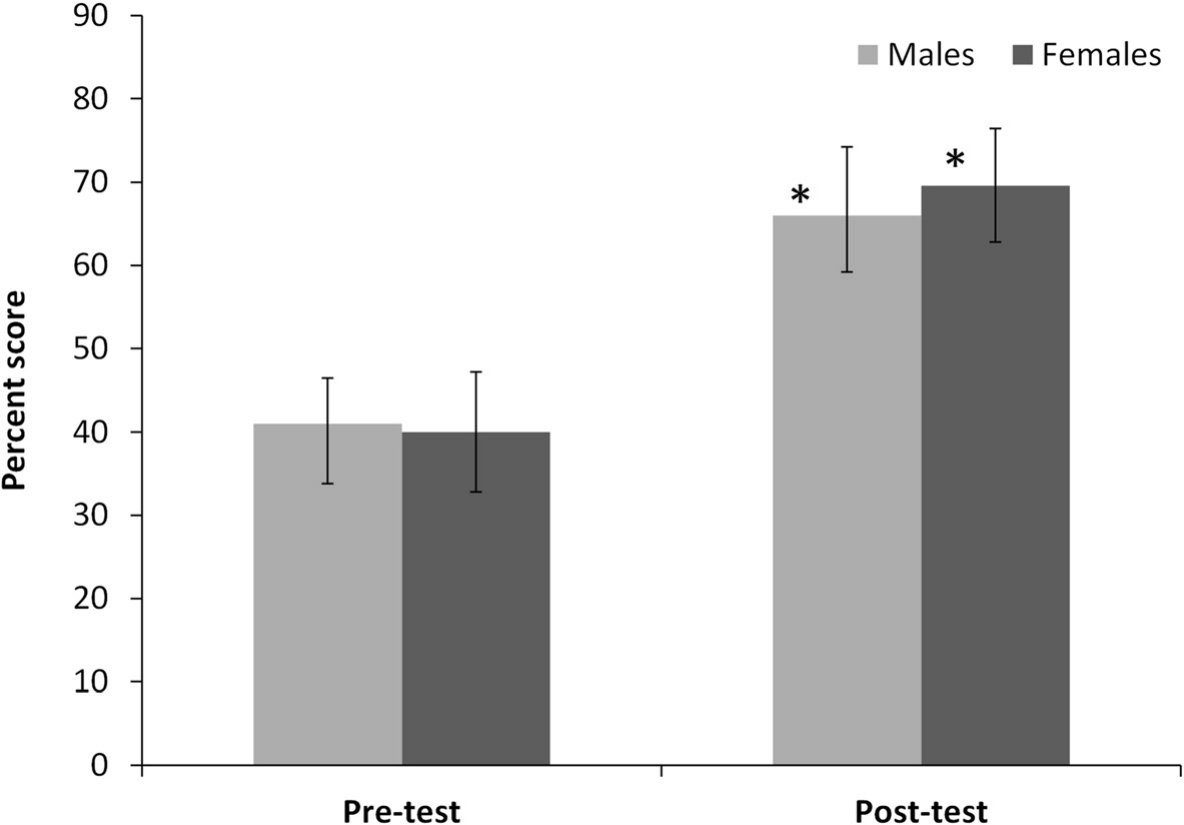
Mean percent scores of males and females in the pre-test and post-test. *Difference from relative pre-test score is significant at *p* < 0.001 by Wilcoxon test. No significant difference was found between males and females on the pre-test (*p* = 0.594) or post-test (*p* = 0.124). Error bars: standard deviation

Of the 29 students, 34.4 % were in their fourth year, 24.1 % in their fifth year, and 41.4 % in their sixth year. Demographics of the participants (age, sex and academic year) are presented in Table 2. To assess the gain in knowledge from the course, the pre-test and post-test results were compared (Fig. 1). The overall mean score was 41.0 % ± 6.29 SD on the pre-test and 67.7 % ± 7.70 SD on the post-test (*p* < 0.0001). There was no significant difference between the mean scores of males and females on the pre-test or on the post-test (Fig. 1).

**Table 2:** Distribution of participants by sex, age and academic year

Academic year	Male		Female		TOTAL	
	n	Mean age (SD)	n	Mean age (SD)	n	Mean age (SD)
4	5	24.0 (4.0)	5	22.3 (0.7)	10	23.1 (3.0)
5	3	23.5 (0.8)	4	23.3 (0.6)	7	23.4 (0.7)
6	5	24.6 (0.7)	5	23.7 (0.2)	10	24.2 (0.7)
TOTAL	15	24.1 (2.4)	14	23.1 (0.8)	29	23.6 (1.9)

Given that the students were from three different years of medical school, we wondered whether the one or two years’ difference in medical school education might affect the scores. But comparison of the pre-test mean scores of students in the fourth, fifth and sixth years showed that there was no significant difference between them (39.2 % ± 6.20 SD, 43.4 % ± 7.80 SD and 41.0 % ± 5.44 SD, respectively; *p* = 0.317). Likewise, there was no significant difference between them on the post-test (data not shown).

Finally, we looked at how the students evaluated the course (Table 3). Most of them (76 %) found it interesting and stated that their personal goals were met by the program (72.4 %). On a Likert scale measuring satisfaction with various aspects of the course (Table 2), the statement “Overall, the instructors were effective and responded to questions in an informative, appropriate and satisfactory manner” received the most positive answers (93.1 % agree or strongly agree). The statement receiving the least positive answers (58.6 %) was “The workshop was scheduled at a suitable time of year.” Many students commented that the course was appropriate and relevant to their medical education. Some students complained about some technical difficulties during videoconferencing or suggested that more time should be given for the community education session. In response to open questions concerning improvement of the curriculum, 4 of 29 students (14 %) expressed interest in an additional week of simulation sessions. In conversation after the simulation sessions, all of them expressed strong interest in XVR and ISS.

**Table 3:** Scores of student satisfaction with course

Statement	% Strongly agree	% Agree	% Positive answers	% Neutral	% Disagree
Overall, the pre-workshop course was appropriate and informative.	37.9	37.9	75.9	17.2	6.9
The workshop was scheduled at a suitable time of year.	44.8	13.8	58.6	17.2	20.7
Overall, the workshop facilities and location were appropriate and satisfactory.	41.4	48.3	89.7	10.3	0.0
Overall, the workshop material was presented in a clear and organized manner.	37.9	41.4	79.3	17.2	3.4
Overall, the instructors were effective and responded to questions in an informative, appropriate and satisfactory manner.	51.7	41.4	93.1	3.4	3.4
Overall, the handouts for discussion groups and case studies were clear and useful.	41.4	34.5	75.9	20.7	3.4
Overall, the workshop was informative and valuable.	34.5	48.3	82.8	17.2	0.0

We also followed up the students by email and telephone one and a half years after the course to get some understanding of their attitudes and experience with disasters after the course. Only 18 students of the 29 (62 %) could be contacted. Half of them were working as interns or residents in the Jazan area, while the rest had transferred to Riyadh, Saudi Arabia. In December 2015, a fire broke out in the maternity ward at Jazan General Hospital, killing 25 and injuring 123, and though none of these 18 students were working at that hospital, they helped prepare their hospitals and participated in receiving the victims and discharging stable cases. They said that the course had changed their attitude and that they have become less stressed and more confident when faced by emergencies at their hospitals. Overall, they said they feel that they had benefited from the course and, for example, could distinguish what was being done improperly during drill evacuations at their hospital.

## Discussion

This study shows that the curriculum developed for Saudi Arabian medical schools is significantly effective in increasing the students’ average knowledge of disaster medicine. The statistically significant increase in overall mean score from 41.0 % on the pre-test to 67.7 % on the post-test is somewhat better than that described in a similar study, which reported that the scores increased from 39 % on the pre-test to 58 % on the post-test [26]. However, our students were selected for having above average grades and good knowledge of English. Nevertheless, this selection represents the real situation of students who would be sent for overseas training if the course is adopted by Saudi Arabian medical schools. The course was conducted at CRIMEDIM, which started international summer course programs for disaster medicine education two years ago. The performance and competences the students gained from this course motivated Jazan University to sign a memo of understanding with CRIMEDIM to include the course in one of the four international programs for medical students at Jazan University.

Though male medical students are separated from female students in Saudi Arabia, they follow the same curricula and the same standards are applied. So it was not surprising that the scores of males and females were not different, either on the pre-test or the post-test. Moreover, given that disaster medicine is not taught in the undergraduate years, the results confirmed our expectation that students in their fourth, fifth or sixth year would perform similarly in the pre-test.

According to the National Educational Framework for disaster health, students can be trained and educated in the basic skills needed for the response to mass casualties [13]. These include preparedness, planning, response and recovery, and understanding the roles of different organizations. The course has a community-based disaster medicine curriculum that encourages learners to apply concepts, skills and attitudes to their unique local context. This course considered the specific needs of the medical students and the community at large and consisted of an appropriate number of learning activities in a balanced variety of educational settings. The community disaster awareness sessions (considered a novel and important topic in Middle Eastern countries) was designed according to health needs assessment in Jazan and aim at providing the students with the basic skills and knowledge required for educating the community at different levels (local population and community members).

In their final three years, medical students can work as volunteers. One study demonstrated that up to 96 % of medical students are willing to volunteer during disasters by helping triage and by providing first aid and community education [10]. In the 2005 earthquake in Kashmir, medical students participated in search and rescue operations and provided emergency care under supervision [27]. Importantly, students who are not properly prepared and protected can cause dangers to themselves as well, and the first issue that should be considered when involving students in emergency response is their safety. In the course, we included sufficient practical training in the use of personal protective equipment. They were also instructed in how to prepare their personal /family disaster plans and oriented in how to approach a disaster scene, particularly when the hazards are not known. Emergency first responders, whether military or civilian personnel, are given priority access to protective equipment or vaccines, and medical students who serve as volunteers should also be given such priority [28]. Because they are still in training and are newly involved in the disaster response, their occupational risk has to be reduced by giving them priority in receiving all types of protection ahead of the qualified individuals in the medical groups [28].

During disasters, healthcare workers are faced by decision-making difficulties in three main areas: triage of the patients, resource allocation, and clinical care. One study showed that triage can be learned by first year medical students with a degree of accuracy that is comparable to that of more experienced peers [29]. We expect that medical students in their fourth to sixth year of medical school can perform equally well if not better. Providing additional human resources for triage can free the specialized professionals for other duties.

This curriculum was developed in the context of Jazan University’s annual international summer school program, which provides for overseas training of 340 students from different colleges. Thus, it was possible to develop a rather extensive curriculum that extends beyond implementation of knowledge for management to aspects of educating peers and the community. The program encompasses more than 53 h of education over 2 weeks of different learning activities structured around five major domains. The complex humanitarian emergency topics, the Sphere project, and techniques to handle psychological reactions caused by disasters are considered new topics in undergraduate programs aiming to help medical students to participate as volunteers in disaster humanitarian relief.

The course was designed to teach knowledge and skills sequentially, in that basic principles are first presented in didactic sessions, and then competencies are strengthened by practicing skills in hands-on exercises and simulation sessions. Then, the acquired knowledge and skills are implemented by designing sessions geared towards community education and disaster management education for their colleagues, such as medical and health care students at universities. We also foresee the possibility of recruiting students from other health disciplines. Inter-professional learning with their future colleagues eventually improves the quality of care and assistance provided during a disaster [30]. For example, involvement of medical students with other health care students (nurses, laboratory technicians) helps the students to know the roles and responsibilities of other health care workers and at the same time provides them with greater understanding of teamwork and communication skills in complex patients care situations [31–33].

All the learning activities are inter-related, and this creates a framework for effective disaster medicine training [34]. It has been demonstrated that participants in courses using different teaching methods are more confident in their knowledge for at least 6 months following the training [35].

The gold standard for demonstrating the effectiveness of a curriculum in medical education is the demonstration of a change in behavior or performance in the real-world clinical setting. However, it is often more feasible to measure changes in knowledge and educational satisfaction, [17, 36, 37] which was done in this study. The students’ experience encompasses important factors such as satisfaction with teaching and perception of quality of the learning material, physical environment, and learning culture [38]. The course evaluation process helps guide future modifications of the curriculum in order to better meet the program’s objectives [39]. In addition to evaluating the course by comparing the pre-test and post-test scores, we sought feedback from students. This feedback was quite positive, and so was the less formal follow-up one and a half years after the course.

This is the first interventional study that focuses on the education of medical students in Saudi Arabia by using an internationally developed, approved and delivered curriculum. On the level of the Gulf region and other Arab countries, this is the first competency-based disaster medicine curriculum focused on blended learning and simulation-based exercise, and nothing was found in the literature about similar programs in Arab countries. On the other hand, though information can be found about community based programs in the literature, [40] there is not much on providing the students with the skills, knowledge and attitude required for community awareness.

One major challenge in this study was the budgetary constraint, which limited the number of participants. Further studies should enroll larger numbers of students, and expenses should be seen as an investment rather than a cost. The extent and depth of the course enables the course graduates to educate others, and in that, the course trains trainers. Establishment of appropriate courses in disaster medicine in Saudi Arabia in which course graduates train students in medicine and other health professionals will increase the capacity and competence for dealing with disasters and will generate a return on the investment.

## Conclusion

This study demonstrates the efficacy of the proposed course in disaster medicine for Saudi Arabian medical students. This course would help to increase the size of the human resources available for dealing with disaster situations. Moreover, graduates of this course can be integrated in teaching disaster medicine to other students in medicine and other health disciplines. This course could be adapted to other countries in the region by replacing the components that have been adapted to the Saudi Arabian context with others that are relevant to the target society.
